# Expression characteristics, molecular mechanisms, and clinical significance of DICER1 in breast cancer

**DOI:** 10.3389/fgene.2025.1586287

**Published:** 2025-07-01

**Authors:** Xi Zhang, Long Yu, Cuizhi Geng

**Affiliations:** ^1^ Department of Breast Center, The Fourth Hospital of Hebei Medical University, Shijiazhuang, Hebei, China; ^2^ Department of Anaesthesia, The Fourth Hospital of Hebei Medical University, Shijiazhuang, Hebei, China

**Keywords:** breast cancer (BrCa), DIcer1, tumor mutation burden (TMB), prognostic biomarker, immune microenvironment, drug sensitivity

## Abstract

**Objective:**

This study aims to investigate the expression patterns, molecular mechanisms, and clinical significance of DICER1 in breast cancer (BRCA), providing new biomarkers and therapeutic targets for prognosis assessment and personalized treatment of breast cancer.

**Methods:**

By integrating RNA-seq data, clinical data, and tumor mutation burden (TMB) data from The Cancer Genome Atlas (TCGA) database, as well as single-cell transcriptomic data from the Gene Expression Omnibus (GEO) database, we analyzed the expression characteristics of DICER1 in breast cancer. Weighted gene co-expression network analysis (WGCNA) was used to identify gene modules associated with the breast cancer phenotype, and gene set enrichment analysis (GSEA) was performed to explore their biological functions. Cellular experiments were conducted to verify the effects of DICER1 on the proliferation, migration, and invasion of breast cancer cells. A nomogram model was constructed based on clinical data to evaluate its prognostic value. Additionally, the effects of DICER1 expression levels on drug sensitivity and the tumor immune microenvironment were analyzed.

**Results:**

The expression of DICER1 in breast cancer tissues was significantly lower than that in normal tissues, and was significantly correlated with tumor stage, T stage, and TMB levels. The expression level of DICER1 was an independent prognostic factor for breast cancer patients. The nomogram model based on DICER1 expression and clinical features demonstrated good discriminative ability in predicting patient survival probability. Drug sensitivity analysis revealed that the high-expression group of DICER1 exhibited higher sensitivity to multiple drugs. Immune microenvironment analysis indicated that the low-expression group of DICER1 had higher immune-suppressive features and immune exclusion scores, suggesting potential resistance to immunotherapy. Single-cell transcriptomic analysis revealed heterogeneous expression of DICER1 in breast cancer cell populations and its potential role in cell-cell communication.

**Conclusion:**

DICER1 plays an important regulatory role in breast cancer, with its expression level closely related to tumor progression, the immune microenvironment, and drug sensitivity. DICER1 has the potential to become an important biomarker for prognosis assessment in breast cancer and may provide new targets for future immunotherapy and targeted therapy.

## 1 Introduction

Breast cancer (BRCA) is one of the most common malignant tumors among women globally, with persistently high incidence and mortality rates that pose a significant threat to women’s health (Roy et al., 2023; Michaels et al., 2023). Despite substantial progress in the diagnosis and treatment of breast cancer in recent years, its highly heterogeneous nature and complex molecular mechanisms result in considerable variations in prognosis (Phung et al., 2019; Hill et al., 2018). Therefore, exploring the molecular mechanisms underlying breast cancer and identifying new biomarkers and therapeutic targets are of great significance for improving patient outcomes and facilitating personalized treatment. Among the various molecular pathways involved in breast cancer, the RNA interference pathway has emerged as a promising area of focus due to its critical role in regulating gene expression and its potential to influence tumor development and progression. This study specifically examines the RNA interference pathway regulators, particularly DICER1, to uncover its potential as a novel biomarker and therapeutic target in breast cancer (Zhang et al., 2024; Juul et al., 2010; Fang et al., 2022).

The DICER1 gene is a key enzyme in the RNA interference pathway, responsible for processing precursor microRNAs (pre-miRNAs) into mature miRNAs, thereby regulating gene expression. In recent years, studies have revealed that DICER1 plays an important role in various cancers. For instance, study reported a 17-year-old female patient who had undergone surgery for a mixed SLCT and juvenile granulosa cell tumor at the age of 14, and was later diagnosed with a high-grade sarcoma with rhabdoid differentiation due to pelvic mass recurrence, with the presence of a DICER1 mutation confirmed once again (Shero et al., 2024). Additionally, a literature review summarized several cases involving SLCT and ERMS (embryonal rhabdomyosarcoma) associated with DICER1 syndrome, emphasizing the importance of multimodal treatment approaches, including extensive surgical resection, various chemotherapy regimens, and adjuvant therapies tailored to individual patients (Riascos et al., 2024). However, the specific functions and mechanisms of DICER1 in breast cancer remain to be fully elucidated. Zhang et al. demonstrated that amplification of the MIR191/425 locus is associated with poor survival in breast cancer patients. This miRNA cluster downregulates DICER1 expression by targeting the 3′untranslated region of DICER1 mRNA, thereby affecting global miRNA biogenesis and promoting the proliferation, survival, migration, and invasion of breast cancer cells (Zhang et al., 2018). Moreover, miRNAs from the let-7 family, acting as downstream effectors of the miR-191/425-DICER1 axis, partially counteract the oncogenic effects mediated by miR-191/425. Another study investigated the germline mutations of DICER1 in Chinese patients with familial breast cancer. Although several novel variants were identified, no direct association between these variants and the disease was found, suggesting that DICER1 germline mutations are either rare or absent in Chinese patients with familial breast cancer (Cao et al., 2014).

In recent years, the application of multi-omics analysis technologies has provided new insights into breast cancer research. By integrating genomic, transcriptomic, and epigenomic data, researchers can more comprehensively reveal the regulatory networks of DICER1 in breast cancer and its clinical significance. Moreover, the application of single-cell sequencing and spatial transcriptomics has further elucidated the heterogeneous expression of DICER1 in breast cancer cell populations and its role in immune cell communication. In clinical applications, the expression level of DICER1 has been shown to be significantly correlated with the prognosis of breast cancer patients. Studies have indicated that patients with low DICER1 expression often have poorer survival rates, and its expression level can serve as an independent prognostic biomarker. Additionally, by constructing nomogram models based on DICER1 expression and clinical indicators, researchers can more accurately predict patient outcomes, providing a basis for personalized treatment. In summary, the expression patterns, molecular mechanisms, and clinical significance of DICER1 in breast cancer have become a current research hotspot. Through multi-omics integration and clinical validation, DICER1 not only holds promise as an important biomarker for prognostic assessment in breast cancer but may also provide new ideas for immunotherapy and targeted therapy. However, further large-scale clinical studies are needed to verify its clinical application value and to explore the potential roles of DICER1-related molecules in breast cancer treatment.

## 2 Methods

### 2.1 Data acquisition and sample collection

In this study, we collected bulk RNA-seq data, clinical data (including gender, age, and stage), and tumor mutation burden (TMB) data from 113 normal samples and 1,113 tumor samples from The Cancer Genome Atlas (TCGA) database (https://www.cancer.gov/ccg/research/genome-sequencing/tcga) (Weinstein et al., 2013). Patients with primary BRCA samples of single cell RNA sequencing (scRNA - seq) gene expression data from comprehensive database (GSE248288 dataset (Tan et al., 2024)), contains four BRCA samples (https://www.ncbi.nlm.nih.gov/geo/). We used the GEO dataset GSE131769 (296 tumor samples) for external validation of the column chart model. Additionally, the expression profiles of DICER1 across various cancers were retrieved from the pancancer expression database TIMER2.0 (http://timer.cistrome.org/).

Breast cancer cases initially diagnosed at the Fourth Hospital of Hebei Medical University were included in this study. Patients who had previously undergone surgery, systemic therapy, or radiotherapy were excluded. All patients provided written informed consent for clinical specimen collection (ethical approval number: 2024KY009). Post-operative specimens were fixed in formalin and embedded in paraffin, or preserved in tissue RNA preservative. Two pathologists assessed the pathological sections, and 98 breast cancer specimens were chosen for DICER1 protein expression analysis in cancer tissue. Based on whether tissue samples were immediately preserved in RNA preservative after removal, 39 normal breast tissue and 48 breast cancer specimens were selected for detecting DICER1 mRNA levels in both cancerous and normal breast tissue.

### 2.2 Experimental methods

#### 2.2.1 Cell culture

MDA-MB-231 (CC0301, Cellcook, China) and Hs578t (CC0313, Cellcook, China) cell lines were grown in DMEM containing 10% fetal bovine serum (FBS). Cells were incubated at 37°C under 5% CO_2_, and the medium was refreshed every 2–3 days to maintain viability.

#### 2.2.2 Generation of DICER1-Overexpressing cell lines

Lentiviral vectors for DICER1 overexpression (LPP-H0470-Lv105-A00, H0470) and a control vector (LPP-NEG-Lv105-A00, NEG) were produced by GeneCopoeia (Guangzhou, China). Stable cell lines with DICER1 overexpression (OE-DICER1) and matched controls (Control) were generated via lentiviral transduction. Post-puromycin selection, RNA and protein were isolated to assess DICER1 expression levels.

#### 2.2.3 CCK-8 assay

Two distinct cell lines were plated in 96-well plates, each well containing 1 × 10^3^ cells. Cell proliferation was assessed at intervals of 0, 24, 48, 72, and 96 h by introducing 10 μL of CCK-8 solution into each well, followed by incubation at 37°C for 60 min. Absorbance readings at 450 nm were subsequently taken using a microplate reader (BioTek, Vermont, USA).

#### 2.2.4 Transwell migration and invasion assays

Transwell experiments were conducted using chambers with a diameter of 6.5 mm and pores of 8 μm (Corning, USA). In the invasion test, 1× 10^5^ cells were placed on the upper compartment coated with Matrigel, while the lower compartment contained DMEM enriched with 20% FBS. Following a 24-h incubation, cells were fixed, dyed, and imaged under a microscope (Leica, Wetzlar, Germany), with quantification carried out using ImageJ (version 1.53q). The migration test followed identical procedures, except the upper compartment lacked the Matrigel coating.

#### 2.2.5 Protein analysis by western blot

Whole-cell lysates were prepared using Protein lysate supplemented with a protease inhibitor (20101ES60, Yeasen, China). Proteins were resolved using SDS‒PAGE and transferred to PVDF membranes (Millipore, Billerica, MA, USA). The membranes were probed with primary antibodies targeting DICER1 (F-10, Santa, US, 1:500), β-actin (81115-1-RR, Proteintech, China, 1:5,000). HRP-linked secondary antibodies (S0001, S0002, Affinity, China, 1:10,000) were applied for signal detection, and protein bands were visualized using enhanced chemiluminescence reagents (GBOX EXTENDED, Syngene, United Kingdom).

#### 2.2.6 Real-Time quantitative reverse transcription polymerase chain reaction (RT‒qPCR)

Total RNA was extracted from cells or tissues using TRIzol reagent (Invitrogen) following the manufacturer’s protocol. cDNA was synthesized with the RevertAid First Strand cDNA Synthesis Kit (K1622, Thermo Scientific, US). The expression levels of DICER1, were measured by RT‒qPCR using GoTaq^®^ qPCR Master Mix (A6001, Promega, US), with GAPDH serving as the reference gene. Amplification was performed on an Applied Biosystems StepOnePlus™ Real-Time PCR System (Applied Biosystems, US). Relative mRNA expression was determined using the 2^‒ΔΔCt method. The primer sequences are detailed in Supplementary Table S1.

#### 2.2.7 Immunohistochemistry (IHC)

Paraffin-embedded tissue sections were deparaffinized in xylene and rehydrated with a series of graded alcohols. Antigen retrieval was performed by heating the sections in citrate buffer (pH 6.0) or EDTA buffer (pH 8.0) via a pressure cooker. Endogenous peroxidase activity was quenched with 3% hydrogen peroxide for 20 min, followed by blocking with 5% BSA or serum to reduce nonspecific binding. The sections were then incubated with primary antibodies (1:50) specific to the DICER1 proteins overnight at 4°C. The next day, after washing with PBS, the sections were incubated with a biotinylated secondary antibody via a PV9000 kit (ZSGB-BIO, Beijing, China) according to the manufacturer’s instructions. The signal was visualized using diaminobenzidine (DAB) substrate, resulting in a brown precipitate at the antigen sites. Finally, the sections were counterstained with hematoxylin to visualize the cell nuclei.

### 2.3 Differential expression analysis and tumor mutation burden analysis

In this study, patients with BRCA from TCGA-BRCA cohort were stratified into high and low expression groups based on the expression levels of DICER1. Differential expression analysis was performed on the RNA-seq data of the two groups using the limma algorithm (Ritchie et al., 2015), and 832 differentially expressed genes (DEGs) were identified with criteria of |logFC| > 0.5 and adjusted P-value <0.05 (Supplementary Table S1). The tumor mutation burden (TMB) data for the TCGA-BRCA cohort were obtained from the UCSC Xena database (Goldman et al., 2020). Mutation analysis and mutation landscape visualization were conducted using the “maftools” package in R software (Mayakonda et al., 2018). Gene set enrichment analysis (GSEA) was performed based on the “c5. bp.v7.1. symbols” gene set from the Human MSigDB Collections database (Liberzon et al., 2011), implemented using the “GSVA” package in R software (Hänzelmann et al., 2013).

### 2.4 Weighted gene co-expression network analysis

The gene expression data of the TCGA-BRCA cohort were preprocessed in this study. Data underwent missing value checks, and outliers were identified by clustering analysis of samples. A weighted gene co-expression network was constructed by calculating the adjacency matrix and selecting the optimal soft threshold (β) based on scale-free topology criteria. Modules were identified using the dynamic tree-cutting algorithm and further optimized by merging highly similar modules. The minimum number of genes in each module was set to 30. The relationship between modules and traits was assessed by calculating the correlation between module eigengenes and clinical features (BRCA status). Gene significance (GS) and module membership (MM) values were calculated to identify key genes within each module. Visualization charts, including sample dendrograms, heatmaps, and scatter plots, were generated to display the analysis results. The final outputs included the correlation matrix between modules and traits, GS and MM values, and a list of key genes, which were saved for further analysis.

### 2.5 Immune analysis

The differences in tumor microenvironment (TME) immune cell types, immune suppression features, immune exclusion features, and immunotherapy biomarkers between high and low-risk groups were analyzed using the “IOBR” package in R software (Zeng et al., 2021). Single-sample GSEA (ssGSEA) analysis was performed on the high and low expression groups using the “GSVA” package in R software to deconvolute the abundance of various immune cell infiltrations and immune function scores in tissues. Finally, the TCIA scores of BRCA samples in the high and low expression groups were obtained from The Cancer Imaging Archive (TCIA) database (Clark et al., 2013) to analyze the differences in immunotherapy response between the two groups of patients.

### 2.6 Independent prognostic analysis

Univariate and multivariate Cox regression analyses were conducted to identify independent prognostic factors from the clinical features of BRCA and the molecular expression of DICER1. Subsequently, a nomogram model was constructed using prognostic genes and independent prognostic factors, calibration curves were plotted, and ROC analysis was performed using the “rms” package in R software.

### 2.7 Drug sensitivity and immune microenvironment analysis

Drug sensitivity analysis was performed on the high and low expression groups using the “oncoPredict” package in R software (Author anonymous, 2012) and the GDSC2 database. Drugs with significant differences in IC50 values between the two groups were screened, with a P-value threshold of 0.0000000001. At the same time, we selected the top four drugs identified through the drug sensitivity analysis for molecular docking with DICER1 to evaluate the binding affinity between DICER1 and these anticancer drugs, further supporting the potential of DICER1 as a therapeutic target. The 3D structures of small-molecule compounds were obtained from the PubChem database, and protein structure data of genes were retrieved from the Protein Data Bank (PDB). Finally, drug molecular docking analysis was conducted using the CB-Dock2 online analysis platform (https://cadd.labshare.cn/cb-dock2/php/index.php).

### 2.8 Single-cell RNA-Seq data analysis

The “Seurat” package (Butler et al., 2018) was used to process single-cell RNA-seq data in this study. Raw count data were loaded into Seurat objects, and quality control (QC) was performed based on the number of detected genes (nFeature_RNA), total number of molecules per cell (nCount_RNA), and proportion of mitochondrial genes (percent.mt). Cells meeting the following criteria were retained: nCount_RNA between 1,000 and 100,000, nFeature_RNA between 200 and 7,500, and percent. mt not exceeding 20%. The filtered data were normalized using the LogNormalize method and scaled. Dimensionality reduction was performed by principal component analysis (PCA), and batch effects were corrected using the Harmony algorithm (Korsunsky et al., 2019), followed by clustering analysis using the Louvain algorithm. Cell types were annotated using the SingleR package (Aran et al., 2019).

Cell-cell communication was further analyzed using the “CellChat” package (Jin et al., 2021). Immune cells with high-risk features were filtered from the Seurat object, and expression matrices and cell type annotations were extracted to create CellChat objects. Potential ligand-receptor interactions were defined using the CellChatDB.human database, and the analysis was limited to “secretion signaling” interactions. Expression data were preprocessed to identify overexpressed genes and interactions, which were then mapped to the human protein-protein interaction (PPI) network. Communication probabilities were calculated to infer cell-cell communication networks, and interactions supported by at least 3 cells were filtered out. The number and weight of cell-cell communication interactions were displayed using circular plots, and specific cell type communications were shown using bubble plots. Network centrality scores were calculated to identify major signaling roles of cell types and visualized using heatmaps.

To evaluate the expression of DICER1 and its association with cell phenotypes, the area under the curve (AUC) score of gene sets was calculated using the “AUCell” package (Aibar et al., 2017). The processed Seurat object was loaded, and the AUC score for each cell was calculated using the AddModuleScore function. Cells were classified into “high-expression cell clusters” and “low-expression cell clusters” based on the median AUC score. To identify enriched pathways associated with high and low expression cell clusters, GSEA analysis was performed using the “fgsea” package. Differential expression analysis was conducted between groups using the FindMarkers function, and the resulting gene markers were sorted by log2 fold change and used as input for GSEA. Enrichment analysis was performed using KEGG pathways from the MSigDB database. The top 20 enriched pathways were displayed using bar plots, highlighting pathways with significant enrichment scores (p < 0.05).

### 2.9 Statistical analysis

For the experimental part, statistical evaluations were conducted using GraphPad Prism 10.1.2. Immunohistochemistry (IHC) images were processed using Image-Pro Plus (IPP) 6.0, with mean optical density (MOD) derived by dividing integrated optical density (IOD) by the positively stained region. Results are reported as means ± standard deviations (SDs). Differences between two groups were analyzed using a two-tailed t-test, assuming the data met the test’s prerequisites. A p-value below 0.05 was considered statistically significant. For the analysis part, t-tests were used to compare the differences in DICER1 expression between different clinical groups. The Wilcoxon rank-sum test was employed to compare differences between high and low expression groups. Spearman correlation coefficients were used to assess the correlations between data. All statistical analyses were performed using R software version 4.1.0.

## 3 Results

### 3.1 Experimental validation and bioinformatics analysis of DICER1

**FIGURE 1:**
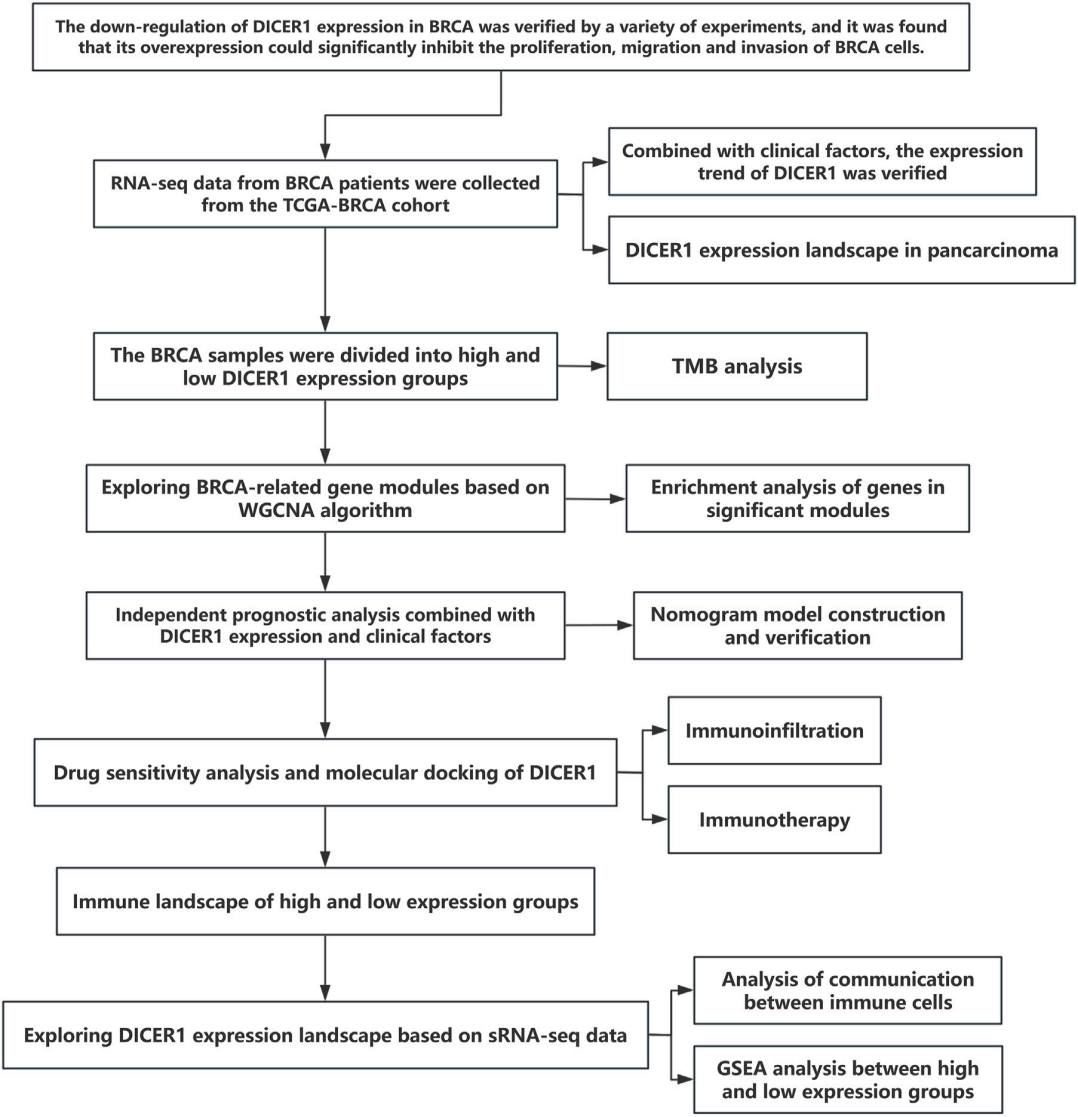
Flowchart of study design and experimental procedures.

**FIGURE 2:**
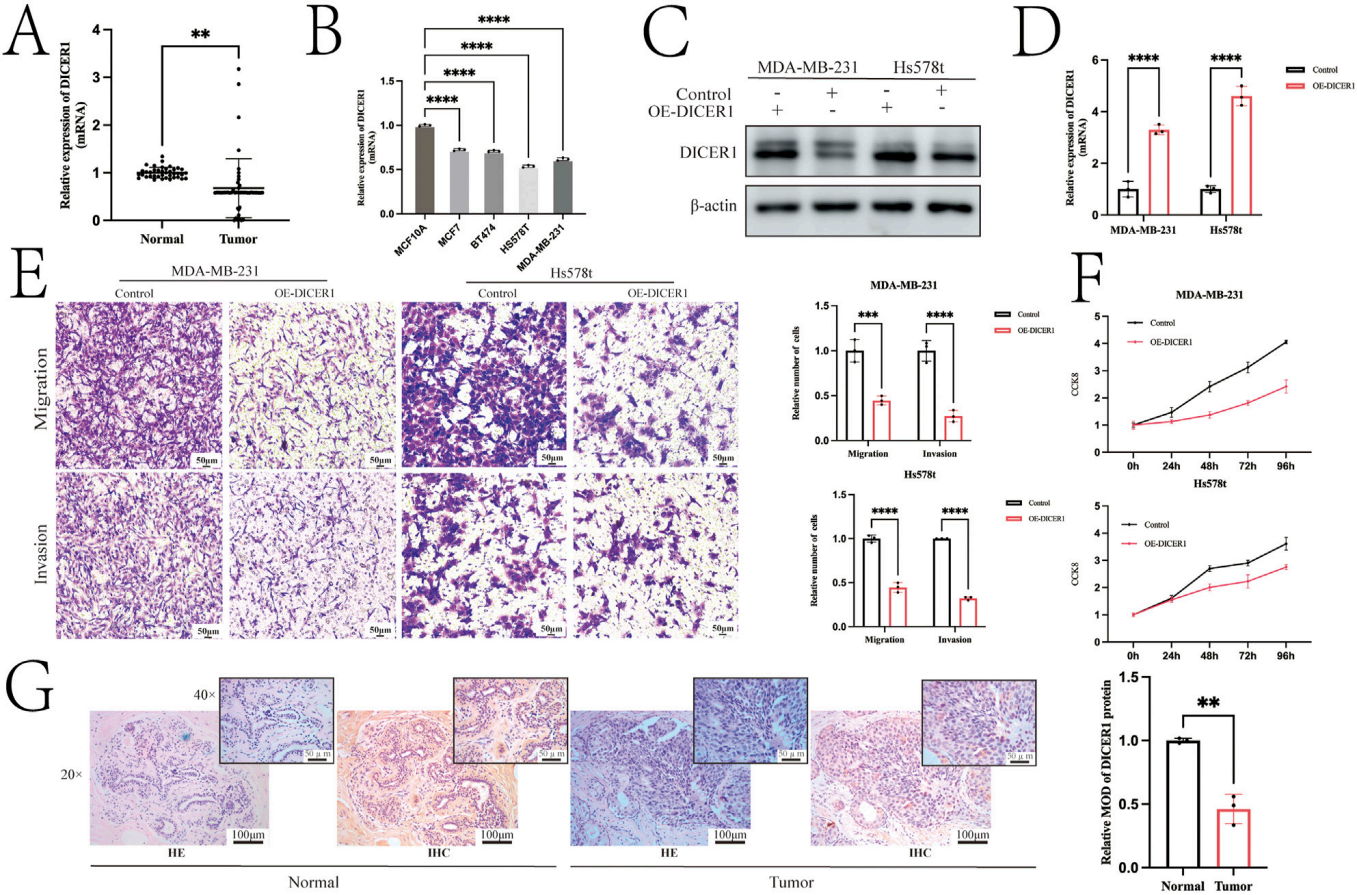
Overexpression of DICER1 inhibits tumor cell proliferation, migration, and invasion in TNBC cells. **(A)** Comparison of DICER1 gene expression levels between breast cancer tissues and adjacent normal tissues. Samples included 39 normal tissues and 48 breast cancer tissues. **(B)** DICER1 mRNA expression levels in different breast cancer cell lines. **(C,D)** Lentivirus was used to construct DICER1-overexpressing cell lines, and overexpression efficiency was verified by Western blot and RT–qPCR. **(E)** Transwell chamber assays demonstrated that DICER1 overexpression inhibited the migration and invasion abilities of breast cancer cells (MDA-MB-231 and Hs578t). **(F)** CCK-8 proliferation assays showed that DICER1 overexpression suppressed the proliferation of breast cancer cells (MDA-MB-231 and Hs578t). **(G)** Immunohistochemistry revealed lower expression of DICER1 in breast cancer tissues compared to normal breast tissues. Data are presented as mean ± SD, n = 3. Significance was determined by two-tailed unpaired t-test; *P < 0.05, **P < 0.01, ***P < 0.001, ****P < 0.0001.

Figure 1 presents the technical roadmap of this study. Total RNA was extracted from normal breast tissues and breast cancer tissues, and DICER1 expression was detected via RT-qPCR. The results indicated that DICER1 expression was significantly lower in tumor tissues compared to normal tissues (P < 0.05, Figure 2A). The expression levels of DICER1 mRNA varied among different breast cancer cell lines, with MDA-MAB-231 and HS578t showing the lowest expression of DICER1 mRNA (Figure 2B). Subsequently, two stable DICER1-overexpressing breast cancer cell lines (MDA-MB-231 and Hs578t) were constructed using lentivirus. Western blot (WB) and RT-qPCR confirmed successful overexpression of DICER1 (P < 0.05, Figures 2C,D). Transwell chamber assays revealed that DICER1 overexpression significantly inhibited the migration and invasion abilities of breast cancer cells (P < 0.05, Figure 2E). CCK-8 proliferation assays demonstrated that DICER1 overexpression markedly suppressed the proliferative capacity of breast cancer cells (P < 0.05, Figure 2F). Immunohistochemical (IHC) analysis further confirmed the downregulation of DICER1 protein expression in breast cancer tissues compared to normal breast tissues (P < 0.05, Figure 2G). Table 1 presents the correlation between clinical characteristics and DICER1 expression in 98 breast cancer patients. DICER1 expression was categorized into low and high groups. The clinical features examined included age, tumor size, postoperative lymph node metastasis status, AJCC tumor staging, molecular typing, and menstrual status. Statistically significant associations were found between DICER1 expression and tumor size (P = 0.002), AJCC tumor staging (P = 0.043), and molecular typing (P = 0.028). No significant correlations were observed between DICER1 expression and age (P = 0.121), postoperative lymph node metastasis status (P = 0.794), or menstrual status (P = 0.209). Notably, in terms of molecular typing, the distribution of luminal A, luminal B, HER2 - positive, and TNBC subtypes differed significantly between the low and high DICER1 expression groups.

**TABLE 1:** Correlation between clinical characteristics and DICER1 expression.

Clinical characteristics	Total	DICER1		*P*
		Low	High	
Total	98	49	49	
Age				0.121
≤45	16 (16.33%)	10 (20.41%)	6 (12.25%)	
45–60	38 (38.78%)	22 (44.90%)	16 (32.65%)	
≥60	44 (44.89%)	17 (34.69%)	27 (55.10%)	
Tumor size				0.002
T1	37 (37.76%)	12 (24.49%)	25 (51.02%)	
T2	57 (58.16%)	36 (73.47%)	21 (42.86%)	
T3	3 (3.06%)	0 (0%)	3 (6.12%)	
T4	1 (1.02%)	1 (2.04%)	0 (0%)	
Postoperative lymph node metastasis status				0.794
N0	69 (70.41%)	33 (67.35%)	36 (73.47%)	
N1	25 (25.51%)	13 (26.53%)	12 (24.49%)	
N2	3 (3.06%)	2 (4.08%)	1 (2.04%)	
N3	1 (1.02%)	1 (2.04%)	0 (0%)	
AJCC tumor staging				0.043
I	22 (22.45%)	7 (14.29%)	15 (30.61%)	
II	73 (74.49%)	39 (79.59%)	34 (69.39%)	
III	3 (3.06%)	3 (6.12%)	0 (0%)	
molecular typing				
luminal A	29 (29.59%)	9 (18.37%)	20 (40.82%)	0.028
luminal B	34 (34.69%)	18 (36.73%)	16 (32.65%)	
HER2 positive	12 (12.25%)	7 (14.29%)	5 (10.20%)	
TNBC	23 (23.47%)	15 (30.61%)	8 (16.33%)	
menstrual status				0.209
premenopausal	36 (36.73%)	21 (42.86%)	15 (30.61%)	
postmenopausal	62 (63.27%)	28 (57.14%)	34 (69.39%)	

**FIGURE 3:**
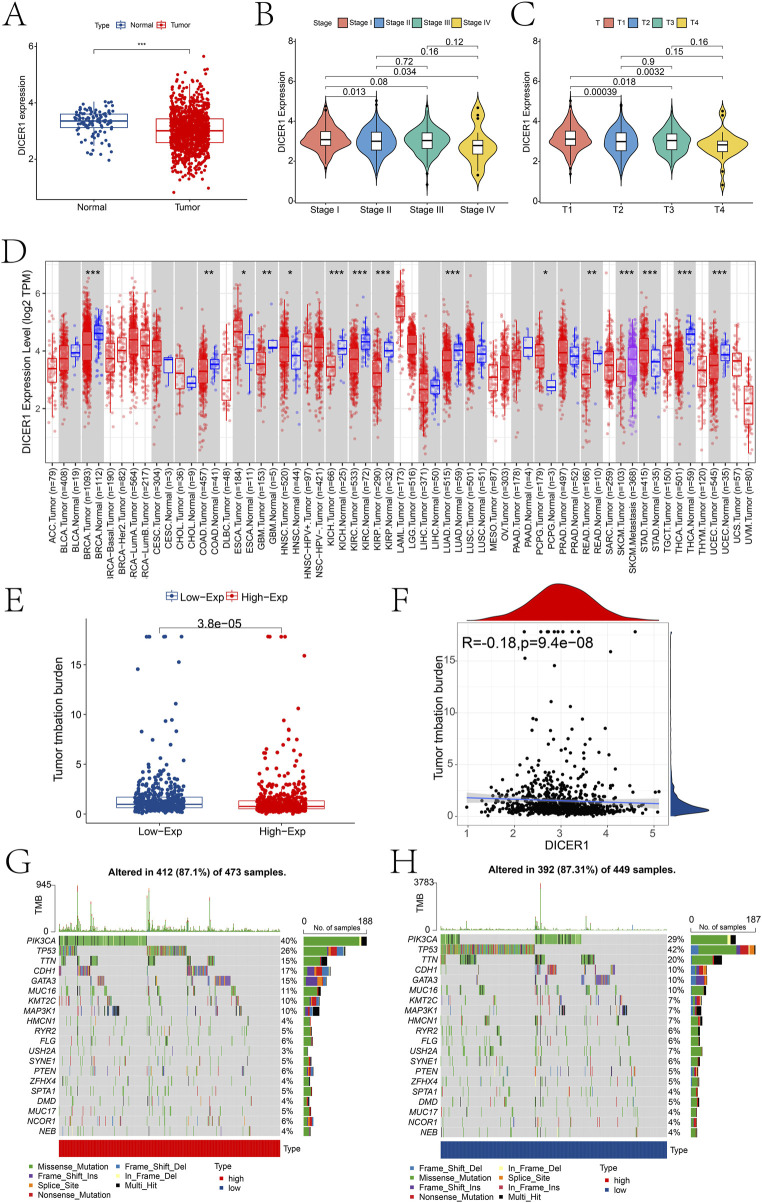
Pan-cancer expression analysis of DICER1. **(A)** Boxplot showing the expression differences of DICER1 between the control and BRCA groups in the TCGA-BRCA cohort. **(B)** Expression differences of DICER1 across different tumor stages. **(C)** Expression differences of DICER1 across different T stages. **(D)** Differences in TMB between high and low DICER1 expression groups. **(E)** Scatterplot of the correlation between DICER1 expression and TMB in high and low expression groups. **(F**–**H)** TMB landscapes of high and low DICER1 expression groups, respectively. *P < 0.05, **P < 0.01, ***P < 0.001.

Bioinformatics analysis of DICER1 expression in the TCGA-BRCA cohort revealed significant differences in DICER1 expression between normal and tumor tissues, as well as across different tumor stages (Stage I-IV) and T stages (T1-T4). In the TCGA-BRCA cohort, DICER1 expression was significantly downregulated in tumor tissues compared to normal tissues (Figure 3A). We analyzed the difference of DICER1 expression among different breast cancer subtypes based on the UALCAN database. The results indicate that there are significant differences in the expression of DICER1 between subtypes such as HER2Pos and Luminal. Detailed information can be found in Supplementary Material 1. Additionally, DICER1 expression exhibited significant differences across various tumor stages (Figure 3B) and T stages (Figure 3C) (P < 0.05). We presented the expression differences of DICER1 between various tumors and their corresponding normal tissues (Figure 3D). Further analysis showed that tumor mutation burden (TMB) differed significantly between high and low DICER1 expression groups (Figure 3E), with a negative correlation between DICER1 expression levels and TMB (Figure 3F; R = –0.18, P = 9.4e-08). The TMB landscape plots (Figures 3G, H) revealed distinct mutation profiles between the high and low DICER1 expression groups, with certain mutation types occurring more frequently in the low expression group. These results suggest that DICER1 may function as a tumor suppressor by modulating TMB and thereby influencing genomic stability in various cancers.

### 3.2 WGCNA analysis and module functional annotation in high- and low-expression groups

**FIGURE 4:**
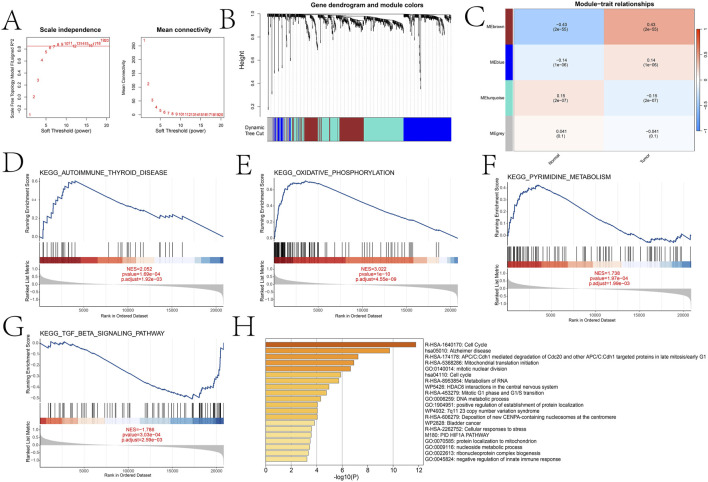
WGCNA analysis comparing key gene modules between high and low expression groups and functional enrichment analysis of different groups and key gene modules. **(A)** Selection of soft threshold power based on scale-free topology fitting (signed *R* ^2^) and average connectivity. **(B)** Dendrogram of gene clustering using hierarchical clustering (average linkage method) based on the dissimilarity of the topological overlap matrix (TOM). **(C)** Heatmap of the correlation between module eigengenes (ME) and traits (normal and tumor). **(D**–**G)** Significant pathways identified by GSEA analysis between high and low expression groups (oxidative phosphorylation, autoimmune thyroid disease, pyrimidine metabolism, and TGF-β signaling pathway). **(H)** Metascape analysis results of the brown module.

We divided patients into high and low expression groups based on the expression of DICER1. Based on the adjusted p-value less than 0.05, we identified the differentially expressed genes of 832 between the high and low expression groups of DICER1 (Supplementary Material 2). Subsequently, we constructed a gene co-expression network by performing Weighted Gene Co-expression Network Analysis (WGCNA) and identified gene modules that are highly correlated with the BRCA phenotype. Based on the scale-free topology model fitting and average connectivity analysis, the optimal soft-thresholding power was determined to be 6 (Figure 4A). Genes were hierarchically clustered using the Topological Overlap Matrix (TOM) dissimilarity measure, and modules were assigned distinct colors (Figure 4B). A total of four modules were identified using the dynamic tree-cutting method. We further explored the relationship between these modules and the BRCA phenotype. The correlation coefficients between module eigengenes (ME) and BRCA are shown in Figure 4C. Notably, the brown module exhibited a strong positive correlation with BRCA (correlation coefficient = 0.43, p = 2 × 10^−55^). To elucidate the differences in biological functions between the high- and low-expression groups, we performed GSEA using the KEGG database. The results revealed several significantly enriched pathways (Figures 4D–G), primarily involved in energy metabolism and cellular respiration. These pathways also implicated immune-related processes and potential functions in nucleotide metabolism. We further explored the functional annotation of genes within the brown module based on the Metascape database (Figure 4H). Genes in this module were significantly enriched in GO terms such as negative regulation of innate immune response and ribonucleoprotein complex biogenesis, suggesting their potential roles in immune regulation and RNA processing. Other modules also showed significant associations with various biological processes, including cell cycle regulation, DNA metabolism, and mitochondrial function.

### 3.3 Independent prognostic analysis

**FIGURE 5:**
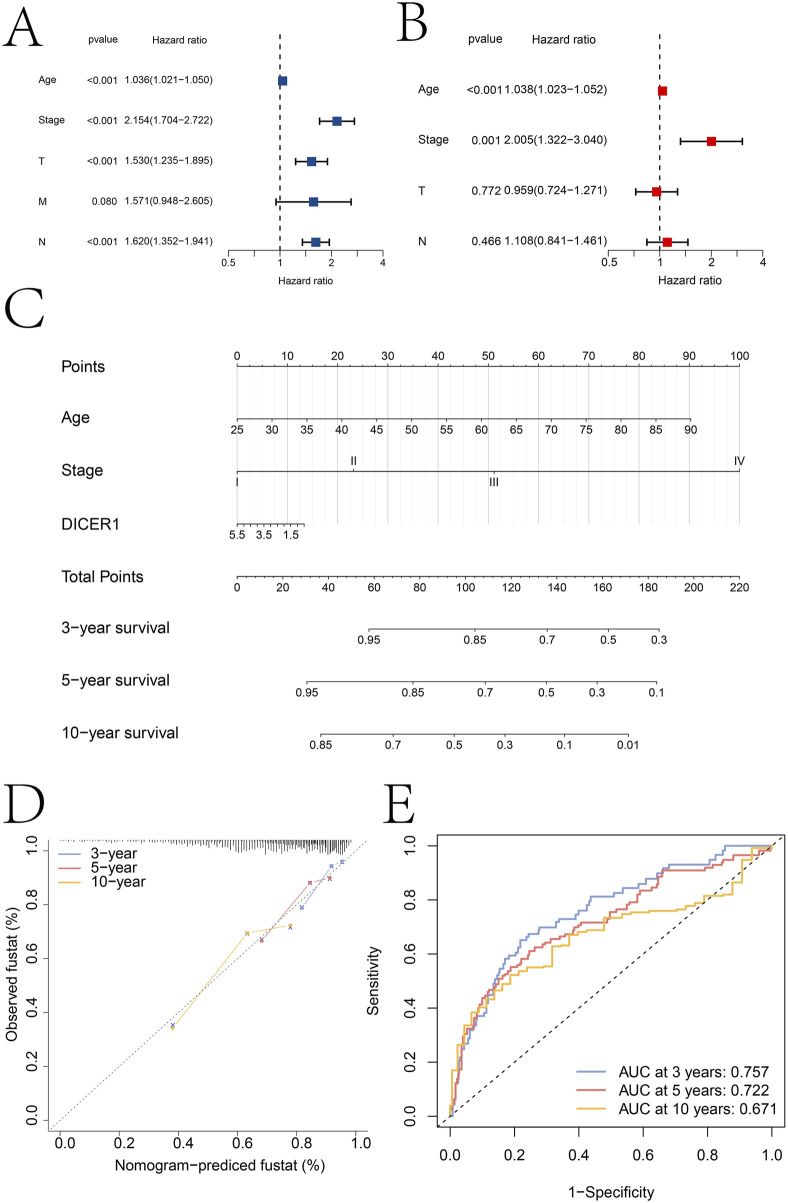
Independent prognostic analysis results. **(A,B)** Identification of independent prognostic factors based on univariate and multivariate Cox regression analyses, respectively. **(C)** Construction of a clinical nomogram model based on age, tumor stage, and DICER1 expression. **(D)** Calibration curve of the nomogram model. **(E)** ROC curves for predicting patient 3-year, 5-year, and 10-year survival using the nomogram model.

Independent prognostic factors were identified through univariate and multivariate Cox regression analyses. In the univariate analysis, age, Stage, T, M, and N were all significantly associated with survival outcomes (Figure 5A). Specifically, the hazard ratios (HR) for age, stage, T, and N were 1.036 (95% CI: 1.021–1.050), 2.154 (95% CI: 1.704–2.722), 1.530 (95% CI: 1.235–1.895), and 1.620 (95% CI: 1.352–1.941), respectively, with all p-values less than 0.001. For M, the HR was 1.571 (95% CI: 0.948–2.605) with a p-value of 0.080. In the multivariate analysis, after adjusting for potential confounding factors, age and stage remained significant independent prognostic factors (Figure 5B). The HRs for age, stage, and DICER1 were 1.038 (95% CI: 1.023–1.052), 2.005 (95% CI: 1.322–3.040), respectively, with all p-values less than 0.001.

Based on the independent prognostic factors identified in the multivariate analysis, a clinical nomogram model was constructed to predict patient survival probability (Figure 5C). The nomogram incorporated age, Stage, and DICER1 as key variables. Each variable was assigned a specific score, and the total score was used to estimate survival probability. The calibration curve of the nomogram showed good consistency between the predicted survival probabilities and the actual observed survival outcomes at 3, 5, and 10 years (Figure 5D). The predictive accuracy of the nomogram was further evaluated using receiver operating characteristic (ROC) curves (Figure 5E). The areas under the ROC curves (AUC) were 0.757 at 3 years, 0.722 at 5 years, and 0.671 at 10 years. These AUC values indicated that the nomogram had moderate to good discriminatory ability in predicting patient survival at different time points. We used the GEO dataset GSE131769for external validation of the nomo model (Supplementary Material S3). The calibration curve analysis results and ROC analysis results show that the nomogram model based on Age, Stage and DICER1 has high accuracy in predicting the 5-year (AUC = 0.764) and 10-year (AUC = 0.624) survival rates of breast cancer patients, which further indicates the generalization of the nomogram model.

### 3.4 Differences in drug sensitivity and immune landscape across groups

**FIGURE 6:**
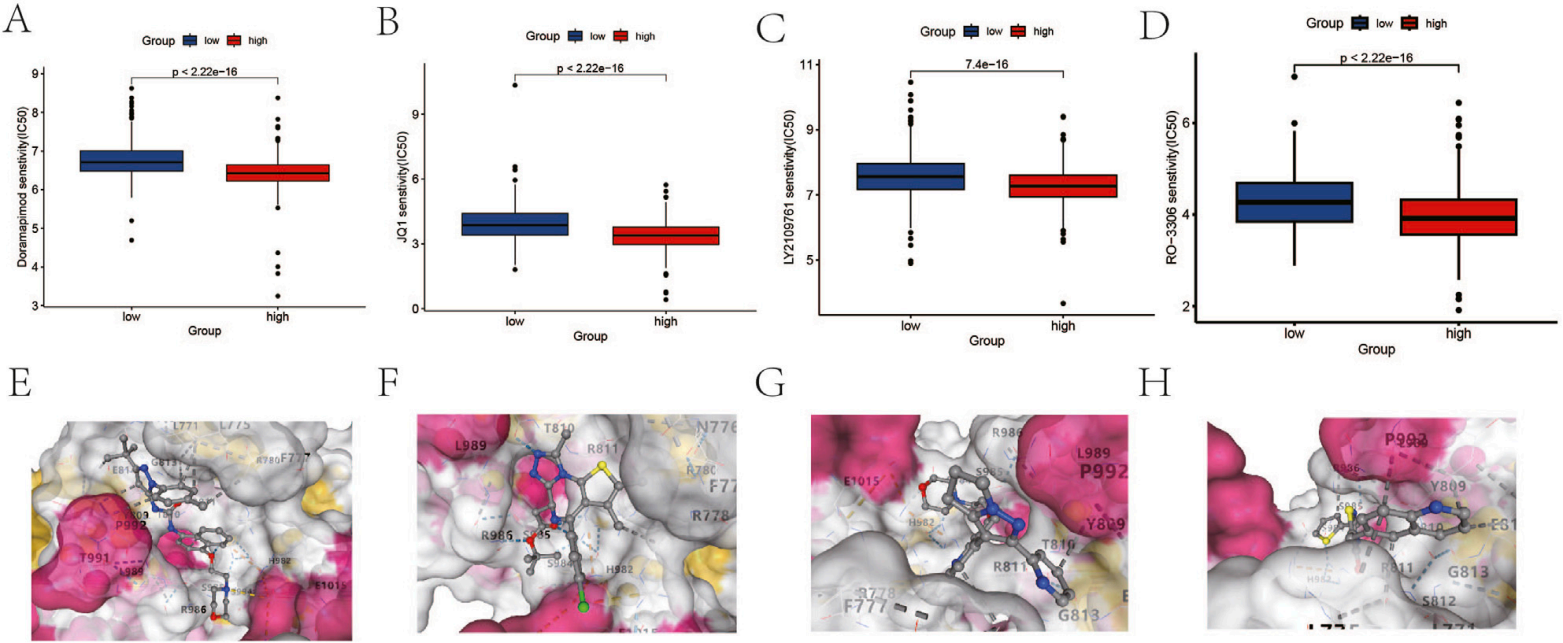
Drug sensitivity analysis and molecular docking analysis based on DICER1 expression levels. **(A–D)** Boxplots of IC50 values for Doramapimod2, JQ1, LY2109761, and Ro-3306 in high and low DICER1 expression groups. The IC50 values of each drug under different expression levels are shown, reflecting the impact of DICER1 expression levels on drug sensitivity. **(E–H)** Molecular docking results of DICER1 with Doramapimod2, JQ1, LY2109761, and Ro-3306. The binding affinity and binding modes of DICER1 with each compound are presented, revealing potential molecular interaction mechanisms.

To further investigate the role of the DICER1 gene in breast cancer, we conducted drug sensitivity analysis and molecular docking analysis to explore the potential of DICER1 in predicting drug response and serving as a potential therapeutic target. The results of the drug sensitivity analysis showed significant differences in the IC50 values of several anticancer drugs between high and low DICER1 expression groups. We selected the top four drugs Doramapimod2, JQ1, LY2109761, and Ro-3306 for presentation. The IC50 values of these drugs were significantly associated with low DICER1 expression (Figures 6A–D). Molecular docking analysis further revealed the binding affinities between DICER1 and Doramapimod2 (−9.8 kcal/mol), JQ1 (−7.9 kcal/mol), LY2109761 (−8.5 kcal/mol), and Ro-3306 (−8.1 kcal/mol), indicating strong binding activities of these compounds with DICER1. These findings suggest that DICER1 may serve as a potential drug target in breast cancer (Figures 6E–H).

**FIGURE 7:**
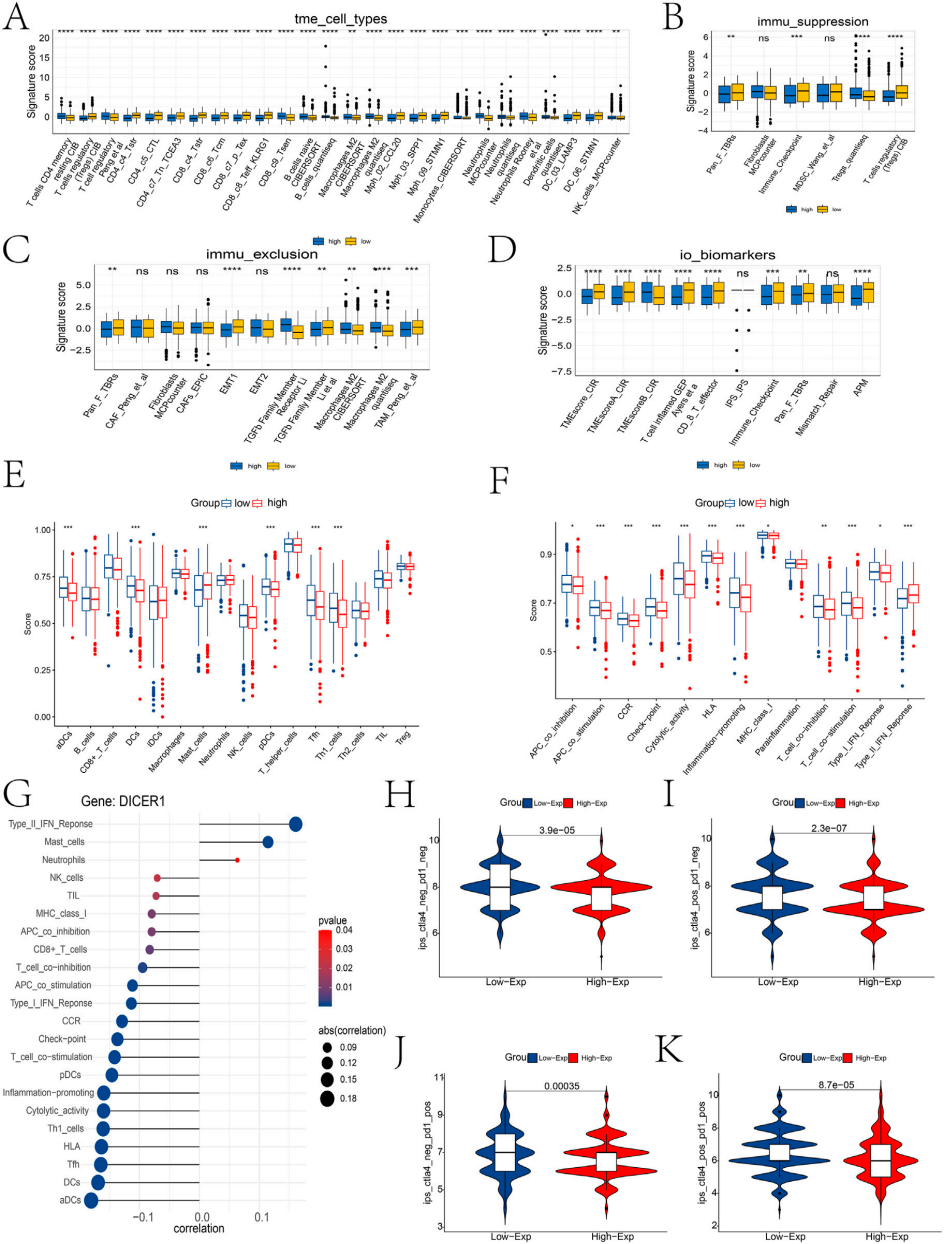
Immune infiltration landscape between high and low expression groups. **(A–D)** Differential analysis of immune cell types, immune suppression features, immune exclusion features, and immune therapy biomarkers in the TME between high and low risk groups using the IOBR package. **(E,F)** Differences in immune cell infiltration abundance and immune function scores between high and low expression groups. **(G)** Lollipop plot of the correlation between DICER1 expression and immune cell infiltration abundance. “Correlation” indicates the correlation coefficient, and p-values indicate statistical significance; the closer the correlation coefficient is to one or -1, the stronger the correlation. **(H–K)** Differential immune therapy responses in high and low risk populations. **(H)** ips_ctla4_neg_pd1_neg group; **(I)** ips_ctla4_neg_pd1_pos group; **(J)** ips_ctla4_pos_pd1_neg group; **(K)** ips_ctla4_pos_pd1_pos group. *P < 0.05, **P < 0.01, ***P < 0.001.

Comprehensive analysis of the tumor microenvironment (TME) revealed distinct immune signatures between high-risk and low-risk groups. Specifically, high-risk patients exhibited higher infiltration levels of T cells, B cells, and dendritic cells, along with significantly elevated levels of Tregs and MDSCs, suggesting a more active yet immunosuppressive immune microenvironment. Additionally, the high-risk group had higher immune exclusion scores, characterized by increased CAFs and TGF-β family members, lower T cell-inflamed gene expression profiles, and higher immune checkpoint expression levels, indicating potential resistance to immunotherapy (Figures 7A–D). We also found significant correlations between DICER1 gene expression and the infiltration of various immune cells and immune function scores (Figures 7E,F), highlighting its potential regulatory role in the tumor microenvironment. The results of the Spearman analysis indicate that DICER1 is significantly correlated with various immune cells or immune functions, including Type II IFN Response, activated dendritic cells (aDCs), dendritic cells (DCs), and inflammation-promoting responses (Figure 7G). Finally, differential immunotherapy response outcomes based on immune checkpoint expression status further underscore the necessity for personalized treatment strategies (Figures 7H–K).

### 3.5 Expression landscape of DICER1 in different cell types

**FIGURE 8:**
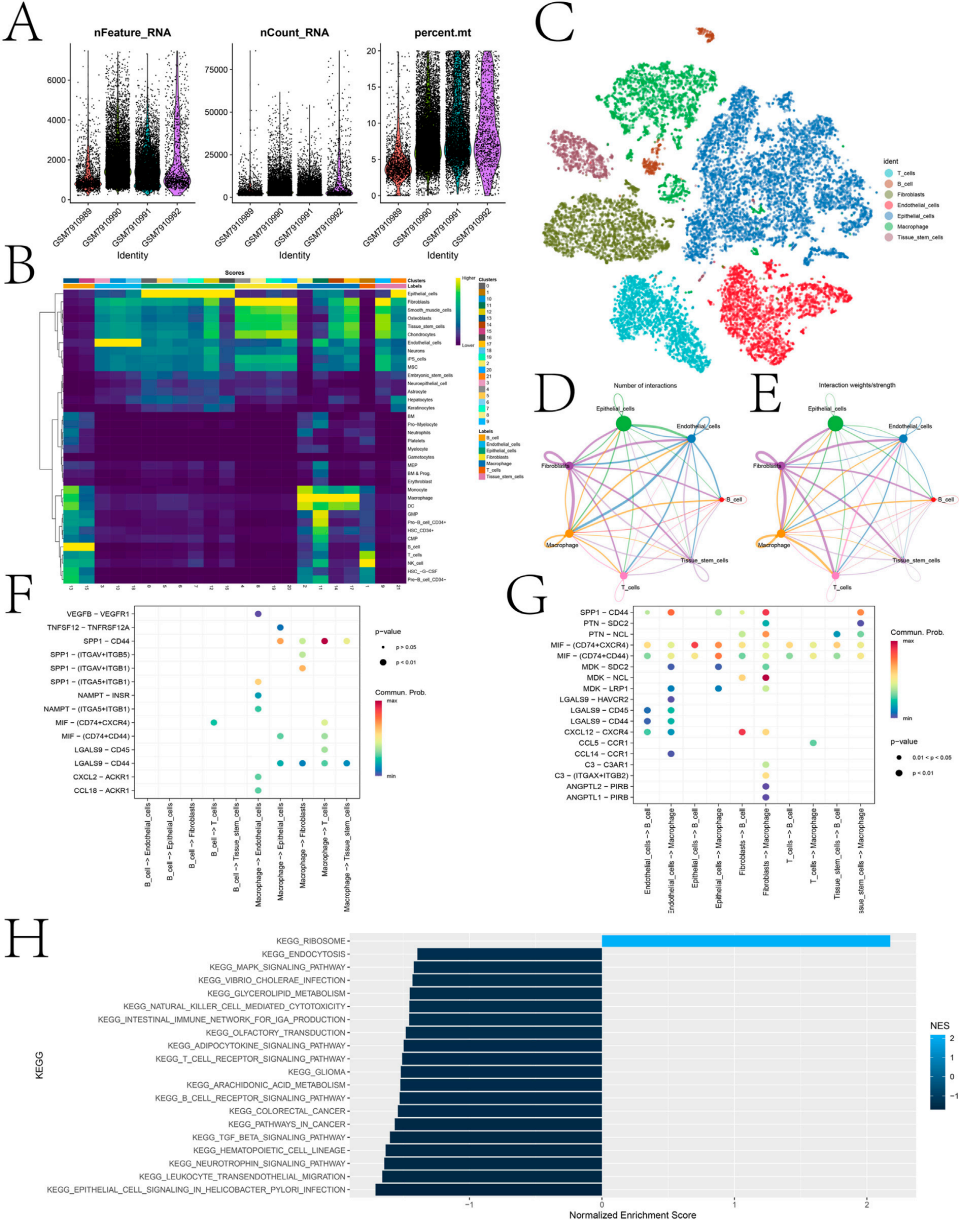
Cell annotation of single-cell sequencing data and expression landscape of DICER1 in different cell types. **(A)** Distribution of multiple quality control metrics after cell quality control. The x-axis represents cluster identity, and the y-axis shows the number of detected RNA molecules (nFeature_RNA) and counts (nCount_RNA) in each cell. The percentage of mitochondrial genes (percent.mt) is also annotated. **(B)** Heatmap showing the expression of key marker genes associated with each cell type. The color scale indicates normalized gene expression levels, with higher values indicating stronger expression. Cell types are listed on the y-axis, and corresponding marker genes are shown on the x-axis. **(C)** Cell clustering and identification based on scRNA-seq data. Each dot represents a single cell, which is grouped into clusters according to its gene expression profile. Clusters are annotated as cell types (e.g., T cells, B cells, fibroblasts, endothelial cells, epithelial cells, macrophages, tissue stem cells). **(D,E)** Interaction networks between different cell types. Arrows indicate the direction of signaling from 1 cell type to another. The thickness of arrows represents the number/strength of interactions, and colors indicate the significance of interactions. **(F,G)** Bubble plots of interaction pathways between immune cells (T cells, B cells, and macrophages) as receptors or ligands with other cell populations. **(H)** GSEA analysis performed on high and low expression cell populations. Bar plots show the normalized enrichment scores (NES) of various KEGG pathways, including those related to cell signaling, immune response, and metabolic processes. The color of the bars indicates the significance of pathway enrichment (p-values), with darker colors indicating more significant enrichment.

scRNA-seq data were subjected to quality control, normalization, dimensionality reduction, and clustering analyses. After quality control, the distribution of multiple metrics across different cell clusters was shown, including the number of detected RNA molecules, the number of features, and the percentage of mitochondrial genes in each cell (Figure 8A). Figure 8B displays a heatmap of key marker gene expression associated with each cell type predicted by the singleR algorithm, highlighting the molecular characteristics of T cells, B cells, fibroblasts, endothelial cells, epithelial cells, macrophages, and tissue stem cells. After annotation, 7 cell types were identified, including T cells, B cells, fibroblasts, endothelial cells, epithelial cells, macrophages, and tissue stem cells (Figure 8C).

Cell-cell communication analysis was performed to elucidate the interaction network among different cell types (Figures 8D,E). Figure 8F,G illustrate the interaction pathways of immune cells (T cells, B cells, and macrophages) as receptors and ligands with other cell populations. Notably, strong and significant communication events were observed in the SPP1-CD44 signaling pathway between macrophages and T cells, and the CXCL12-CXCR4 signaling pathway between fibroblasts and B cells. In this study, all cells were further divided into high and low DICER1 expression groups based on DICER1 expression levels, and GSEA analysis was performed between the two groups (Figure 8H). The enriched pathways included those related to cell signaling, immune response, and metabolic processes.

## 4 Discussion

This study provides a comprehensive investigation into the expression patterns, molecular mechanisms, and clinical significance of DICER1 in BRCA. Through integrative multi-omics analysis, cellular experiments, and clinical data validation, we have elucidated the potential mechanisms of DICER1 in breast cancer and offered novel insights for the prognostic evaluation and personalized treatment of this disease.

We observed a negative correlation between DICER1 expression levels and TMB, yet the exact mechanisms underlying this relationship warrant further in-depth exploration. Although there is currently no direct evidence of DICER1 mutations in breast cancer, studies in other tumor types suggest that DICER1 mutations may contribute to tumor development through haploinsufficiency, which could potentially influence TMB. For instance, in lung cancer research, it has been demonstrated that Dicer1 functions as a haploinsufficient tumor suppressor, with partial loss of Dicer1 promoting tumor development (Kumar et al., 2009). Similarly, in the context of DICER1 syndrome, germline DICER1 mutations have been found to predispose individuals to a range of tumors, with the tumors retaining one functional DICER1 allele, indicating a haploinsufficiency mechanism (Slade et al., 2011). These findings imply that DICER1 mutations, by disrupting miRNA biogenesis, may lead to dysregulation of gene expression and potentially increase the accumulation of other genetic alterations, thereby affecting TMB. However, this hypothesis requires further investigation, particularly in the context of breast cancer, to elucidate the precise relationship between DICER1 and TMB. Additionally, DICER1 is involved in miRNA processing, which can affect DNA repair pathways and genomic stability. For example, DICER1 has been shown to regulate the expression of miRNAs that target DNA repair genes, potentially influencing TMB. In embryonal tumor with multilayered rosettes, a rare but aggressive brain tumor, DICER1 mutations are frequently observed (Lambo et al., 2020). These mutations can lead to biased loading of mature miRNAs, affecting downstream pathways and potentially disrupting the expression of DNA repair genes. In rheumatoid arthritis, DICER1 expression is reduced in fibroblast-like synoviocytes, which is associated with increased inflammation and cellular senescence. This reduction in DICER1 may lead to the accumulation of cytotoxic non-coding RNAs, such as Alu RNAs, which can activate the NLRP3 inflammasome and contribute to chronic inflammation (De Cauwer et al., 2018).

This paper identified gene modules highly correlated with breast cancer phenotypes and revealed their biological functions via GSEA. The brown module showed a strong positive correlation with breast cancer phenotypes, with its genes significantly enriched in pathways related to immune regulation and RNA processing. This indicates that DICER1 may influence breast cancer progression by modulating the immune microenvironment and RNA metabolism. Additionally, we explored the potential links between breast cancer and three pathways: cell cycle, Alzheimer’s disease, and mitochondrial translation initiation. Dysregulation of the cell cycle is commonly associated with breast cancer, characterized by abnormal accumulation of tumor cells (Kashyap et al., 2021). Although Alzheimer’s disease and BRCA are distinct diseases, recent studies have suggested a possible association between them. APOE, a significant risk gene for AD, has been found by Van et al. to potentially prevent chemotherapy-related cognitive decline in elderly breast cancer survivors with the APOE ε2 polymorphism (Van Dyk et al., 2021). In recent years, mitochondrial translation initiation has been identified as closely related to the occurrence, development, and therapeutic response of breast cancer, and it may become an important target for breast cancer treatment (Fuentes-Retamal et al., 2020). Univariate and multivariate Cox regression analyses indicated that DICER1 expression is an independent prognostic factor for breast cancer patient survival. The nomogram model constructed based on DICER1 and clinical features demonstrated moderate to good discrimination in predicting patient survival probabilities, further confirming the potential of DICER1 as a prognostic biomarker.

This study also revealed the impact of DICER1 expression levels on drug sensitivity through drug sensitivity analysis and molecular docking experiments. The high-DICER1 expression group exhibited higher sensitivity to drugs such as AZD7762 and Bortezomib (Figures 6A–D), which may be related to its potential roles in cell cycle regulation and protein degradation. Moreover, immune microenvironment analysis showed that the low-DICER1 expression group had higher immune suppression features and immune exclusion scores (Figures 7A–C), suggesting potential resistance to immunotherapy. These results highlight the complex role of DICER1 in tumor immunity and provide new targets for breast cancer immunotherapy. For example, AZD7762 is a potent ATP-competitive checkpoint kinase inhibitor with potential therapeutic effects in breast cancer, especially triple-negative breast cancer (Zhu et al., 2021). Bortezomib (BTZ) can kill tumor cells via the NF-κB signaling pathway, activate caspase-3 to inhibit cancer-associated fibroblast (CAF) activity, and enhance CD8^+^ T cell function by modulating the expression of immune stimulatory factors, thereby treating BRCA (Liu et al., 2023). It has been found that SB216763 can severely inhibit phospholipid synthesis in BRCA cancer cells (Phyu et al., 2019). WZ4003 is a small-molecule inhibitor with high specificity for epidermal growth factor receptor (EGFR). Recent studies have shown that WZ4003 may play a role in the pathogenesis of breast cancer by inhibiting the EGFR signaling pathway (Li et al., 2022).

This paper revealed the heterogeneous expression of DICER1 in breast cancer cell populations and its potential role in intercellular communication. Cell communication analysis showed significant communication events in the SPP1-CD44 signaling pathway between macrophages and T cells, and the CXCL12-CXCR4 signaling pathway between fibroblasts and B cells (Figures 8F,G). These results suggest that DICER1 may influence the tumor microenvironment by modulating intercellular communication. Additionally, GSEA analysis of high- and low-expression groups identified several significant pathways. For example, Guizhi Fuling Decoction exerts anti-proliferative, pro-apoptotic, and anti-angiogenic effects by modulating the PI3K and MAPK signaling pathways, thereby inhibiting breast cancer (Dai et al., 2020). In triple-negative breast cancer (TNBC), overexpression of MAL2 promotes endocytosis, leading to resistance to novel therapeutic agents. Studies have shown that TNBC patients with high MAL2 expression have a poorer prognosis, with a significantly lower 5-year survival rate (71.33% vs 89.59%, p = 0.0224), independent of PD-1 expression levels and clinical pathological features of the tumor (Borowczak et al., 2024).

Despite revealing the important role of DICER1 in breast cancer, this study has some limitations. First, the sample size is limited, and further large-scale clinical studies are needed to validate the prognostic value and therapeutic potential of DICER1. Second, the specific molecular mechanisms of DICER1 still need to be elucidated through *in vitro* and *in vivo* experiments. Third, experimental validation of immune-related phenotypes, such as immune cell infiltration assays, is missing. Future studies should include these assays to better understand the role of DICER1 in the tumor microenvironment. Additionally, future studies can explore the combined application of DICER1 with other molecular biomarkers to improve the diagnosis and treatment of breast cancer. In summary, through integrative multi-omics analysis and clinical validation, this study elucidated the expression patterns, molecular mechanisms, and clinical significance of DICER1 in breast cancer. DICER1 not only has the potential to become an important biomarker for prognostic evaluation of breast cancer but may also provide new insights for immunotherapy and targeted therapy. However, its clinical application value still needs further validation, and future research should focus on its role in the tumor microenvironment and potential therapeutic targets. Future research should also aim to establish the causal relationship between DICER1 and TMB through additional molecular biology experiments, and validate the tumor-suppressive role of DICER1 and its correlation with TMB in animal models and clinical samples.

## 5 Conclusion

This study comprehensively investigated the expression profiles, molecular mechanisms, and clinical significance of DICER1 in breast cancer by integrating multi-omics analysis, cellular experiments, and clinical data validation. DICER1 was found to be downregulated in breast cancer tissues and closely correlated with tumor stage, tumor mutational burden (TMB) levels, and immune microenvironment features. By constructing a nomogram model, we further confirmed the potential of DICER1 as an independent prognostic biomarker. Moreover, the expression level of DICER1 affects the sensitivity to multiple drugs and may influence the tumor microenvironment through the modulation of intercellular communication. Future studies should further explore the mechanisms of DICER1 in breast cancer and validate its potential application value in clinical therapy.

## Data Availability

The original contributions presented in the study are publicly available. This data can be found here: https://www.jianguoyun.com/p/DUtftjQQ04KmDRiV-vkFIAA.
