# Supplementary material for: Expression characteristics, molecular mechanisms, and clinical significance of DICER1 in breast cancer
Source: Front Genet. 2025 Jul 1;16:1586287. doi: 10.3389/fgene.2025.1586287 (PMC12259429; doi:10.3389/fgene.2025.1586287)
Supplement: Supplementary file 2 [file DataSheet2.docx]

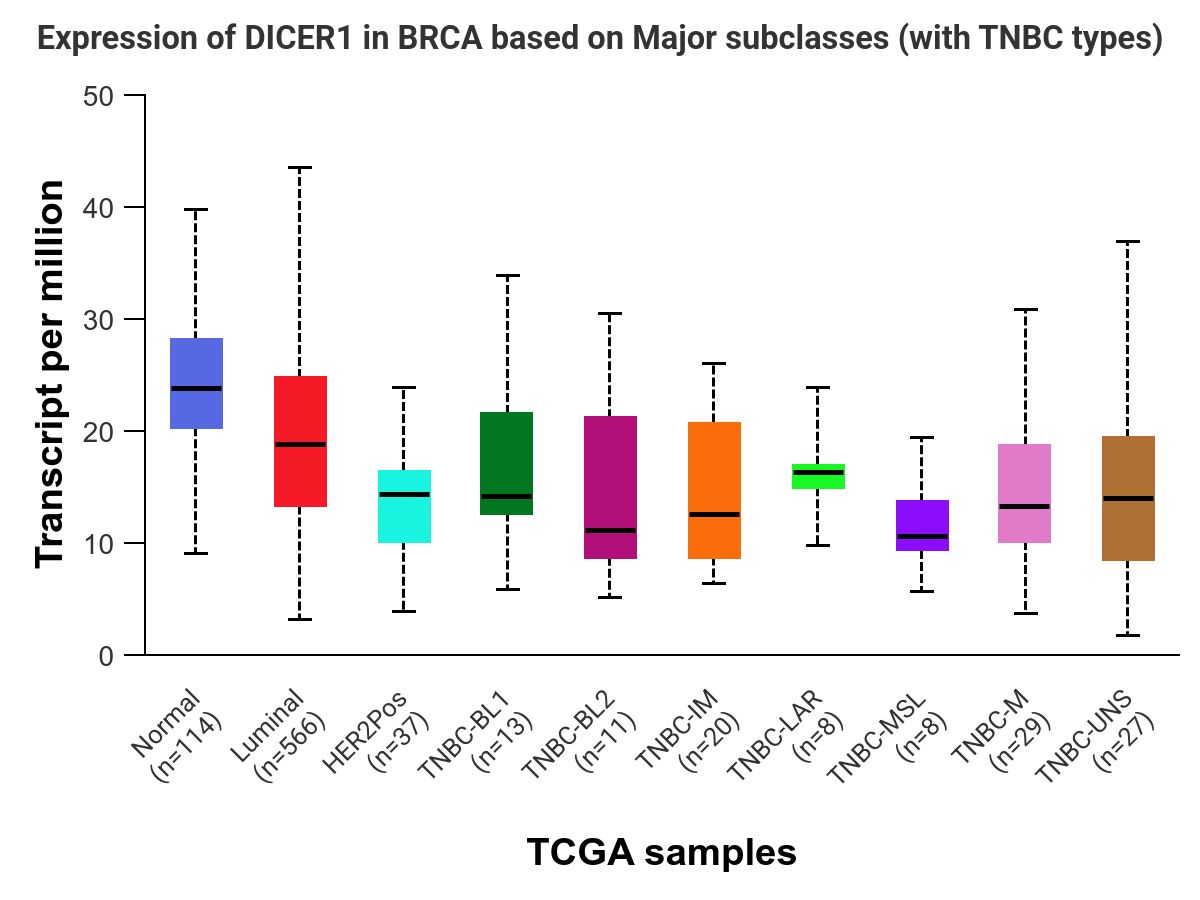


Table Differential expression of DICER1 in different types of breast cancer

| Comparison | Statistical significance |
| --- | --- |
| Normal-vs-TNBC-M | 1.53E-08 |
| Normal-vs-TNBC-IM | 2.39E-06 |
| Normal-vs-HER2Pos | 3.10E-06 |
| Normal-vs-TNBC-UNS | 1.90E-05 |
| Normal-vs-TNBC-MSL | 2.44E-05 |
| Luminal-vs-TNBC-M | 4.45E-05 |
| Normal-vs-TNBC-BL2 | 6.02E-04 |
| Luminal-vs-TNBC-MSL | 6.81E-04 |
| Normal-vs-Luminal | 7.75E-04 |
| Normal-vs-TNBC-BL1 | 1.04E-03 |
| Luminal-vs-TNBC-IM | 1.76E-03 |
| Normal-vs-TNBC-LAR | 1.27E-02 |
| Luminal-vs-HER2Pos | 1.58E-02 |
| Luminal-vs-TNBC-UNS | 2.45E-02 |
