# Supplementary material for: Expression characteristics, molecular mechanisms, and clinical significance of DICER1 in breast cancer
Source: Front Genet. 2025 Jul 1;16:1586287. doi: 10.3389/fgene.2025.1586287 (PMC12259429; doi:10.3389/fgene.2025.1586287)
Supplement: Supplementary file 3 [file DataSheet1.docx]

**Supplementary Materials**


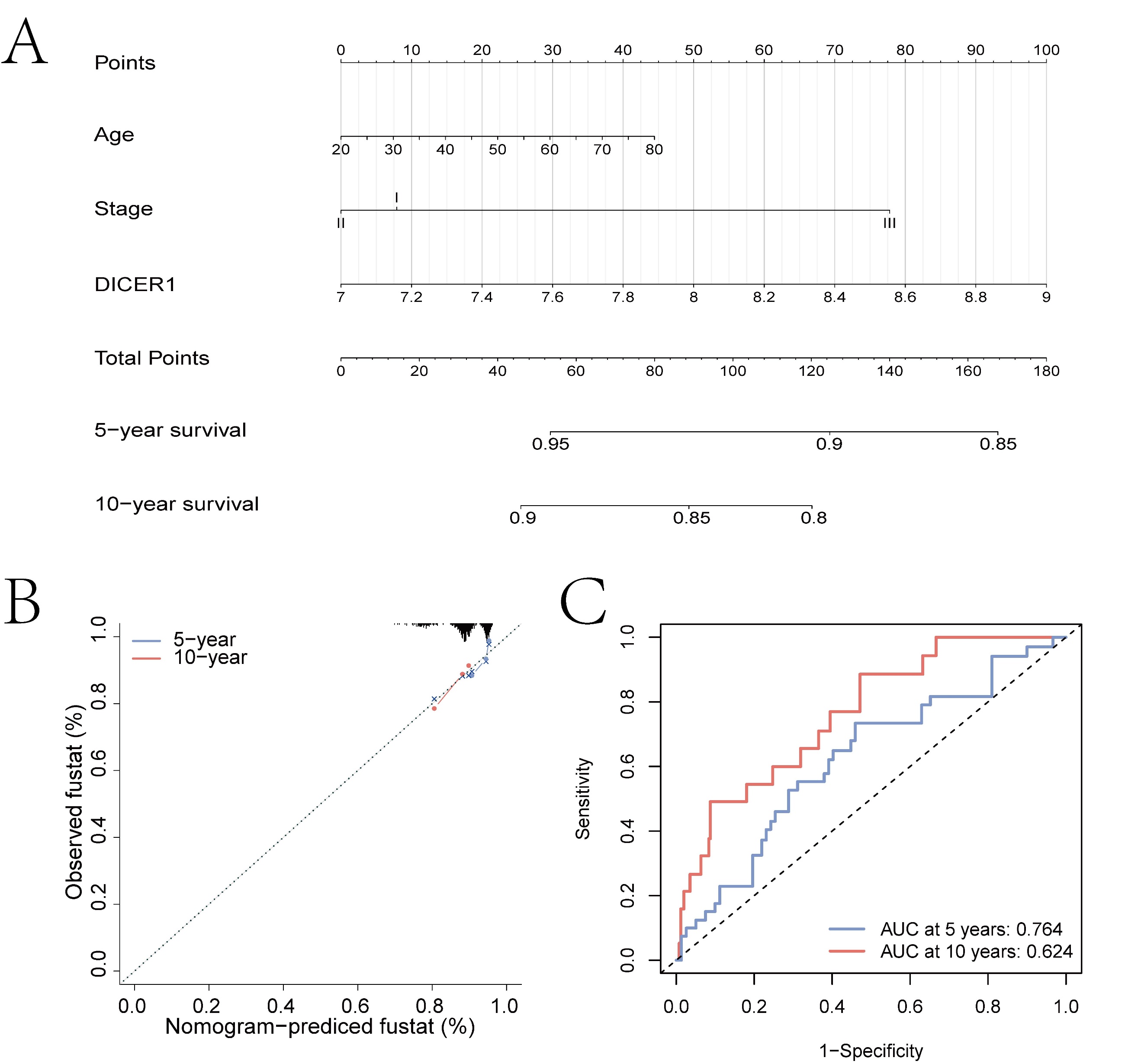


Figure S1 Independent prognostic analysis of DICER1. (A) Nomogram model constructed based on age, stage, and expression level of DICER1. (B)-(C) Calibration curves and ROC curves for predicting 5-year and 10-year survival rates using the nomogram model.
